# RETRACTION: Differential Genetic Mutations and Immune Cell Infiltration in High‐ and Low‐Risk STAD: Implications for Prognosis and Immunotherapy Efficacy

**DOI:** 10.1111/jcmm.70036

**Published:** 2024-08-29

**RETRACTION:** Y.‐Y. Deng, Y. Sun, S.‐J. Wu, T.‐Y. Zhang, J. Yang and K. Liu, ‘Differential Genetic Mutations and Immune Cell Infiltration in High‐ and Low‐Risk STAD: Implications for Prognosis and Immunotherapy Efficacy’, *Journal of Cellular and Molecular Medicine* 28, no. 7 (2024): e18174, https://doi.org/10.1111/jcmm.18174

The above article, published online on 17 March 2024 in Wiley Online Library (wileyonlinelibrary.com), has been retracted by agreement between the Journal Editor‐in‐Chief, Stefan Constantinescu; The Foundation for Cellular and Molecular Medicine; and John Wiley and Sons Ltd. The retraction has been agreed upon as the peer review and the publishing process was found to be manipulated. No evidence of the authors' involvement in this manipulation of the publication process was found. The authors have been informed of the decision to retract but they remained unresponsive.

